# An enriched environment re-establishes metabolic homeostasis by reducing obesity-induced inflammation

**DOI:** 10.1242/dmm.048936

**Published:** 2022-06-13

**Authors:** Sol Díaz de León-Guerrero, Jonathan Salazar-León, Karla F. Meza-Sosa, David Valle-Garcia, Diana Aguilar-León, Gustavo Pedraza-Alva, Leonor Pérez-Martínez

**Affiliations:** 1 Laboratorio de Neuroinmunobiología, Departamento de Medicina Molecular y Bioprocesos, Instituto de Biotecnología, Universidad Nacional Autónoma de México (UNAM), Cuernavaca, Morelos, CP 62210, Mexico; 2 Departamento de Patología, Instituto Nacional de Ciencias Médicas y Nutrición ‘Salvador Zubirán’, Tlalpan, Ciudad de México, CP 14000, Mexico

**Keywords:** Obesity, Enriched environment, Inflammation, Metabolism

## Abstract

Obesity can lead to chronic inflammation in different tissues, generating insulin and leptin resistance and alterations in glucose and lipid metabolism, favoring the development of degenerative diseases, including type II diabetes. Congruently, the inflammatory signaling inhibition prevents the development of obesity and restores insulin sensitivity. Via the enhancement of central nervous system activity, an enriched environment (EE) has beneficial effects on learning and memory as well as on immune cell functions and inflammation in different disease models. Here, we explored whether an EE can restore energy balance in obese mice that previously presented metabolic alterations. We discovered that an EE improved glucose metabolism, increased insulin signaling in liver, and reduced hepatic steatosis and inflammation, and increased lipolysis and browning in the white adipose tissue of high-fat diet (HFD)-fed mice. Finally, we found reduced inflammatory signaling and increased anorexigenic signaling in the hypothalamus of HFD-fed mice exposed to an EE. These data indicate that an EE is able to restore the metabolic imbalance caused by HFD feeding. Thus, we propose EE as a novel therapeutic approach for treating obesity-related metabolic alterations.

This article has an associated First Person interview with the first author of the paper.

## Abstract (precis)

**Summary:** A series of physiological, histochemical and molecular analyses reveal that enriched environment decreases inflammation in adipose tissue and in hypothalamus, re-establishing glucose metabolism in metabolically compromised mice.

## INTRODUCTION

Obesity has become an increasing worldwide health issue over the past years; in some countries it accounts for over 20% of the adult population (OECD, 2017). Obesity is of clinical interest as it is linked with an increased risk of developing other pathologies, such as cardiovascular and neurodegenerative diseases, cancer and type II diabetes (Hotamisligil, 2006). Different studies have shown that obesity is accompanied by a low-grade chronic inflammatory state that has been described in different tissues and organs, including pancreas, skeletal muscle, adipose tissue, liver and brain (Lumeng and Saltiel, 2011). In adipose tissue, an excess in energy intake generates cellular stress, which induces the expression of cytokines and chemokines, leading to the recruitment and activation of different immune cells, mainly macrophages (Gregor and Hotamisligil, 2011). This inflammatory process impairs normal adipose tissue function by inhibiting catecholamine and insulin signaling (Reilly and Saltiel, 2017). In liver, obesity induces lipid accumulation and the activation of different inflammatory pathways that contribute to insulin resistance and a dysregulation in both glucose and lipid metabolism (Koyama and Brenner, 2017). Different studies have shown that an inflammatory process is the determining factor in impairing insulin signaling (de Luca and Olefsky, 2008). Accordingly, inhibition of the inhibitor of nuclear factor kappa (IKK)/nuclear factor κB (NF-κB), Jun-N-terminal kinase (JNK) and inflammasome pathways can prevent weight gain, and restore insulin sensitivity in different obesity models (Hirosumi et al., 2002; Vandanmagsar et al., 2011; Yuan et al., 2001). In this regard, anti-inflammatory therapies have been proposed to treat type II diabetes (Goldfine and Shoelson, 2017).

Recently, this inflammatory process has also been described in the brain. The hypothalamus is the brain region responsible for regulating energy balance by sensing the nutritional state of the body (Remmers and Delemarre-van de Waal, 2011). In hypothalamus, high levels of insulin and leptin activate anorexigenic pathways, while inhibiting orexigenic signaling, to decrease food intake and increase energy expenditure (Morton et al., 2006). Both genetic and diet-induced obesity (DIO) generate an inflammatory process in hypothalamus, characterized by activated JNK and IKK/NF-κB pathways, as well as by increased expression of inflammatory cytokines (Belgardt et al., 2010; De Souza et al., 2005; Zhang et al., 2008). This inflammatory process leads to leptin and insulin resistance, and even neuronal death, impairing the capacity of the hypothalamus to maintain homeostasis (Jais and Brüning, 2017). Nevertheless, studies have shown that the deletion of JNK1 and IKKβ in neurons can rescue leptin and insulin signaling in the hypothalamus, as well as reduce weight gain and restore the metabolic alterations linked to obesity (Belgardt et al., 2010; Sabio et al., 2010; Zhang et al., 2008).

Looking for therapeutic strategies that could have an effect on the brain and the inflammatory response, we decided to study an enriched environment (EE) paradigm. An EE consists of housing conditions that promote increased cognitive, sensory and motor stimuli, as well as social interaction, which lead to the activation of different brain regions (Nithianantharajah and Hannan, 2006). The effects of an EE on brain function have been widely studied and include improved learning and memory, increased long-term potentiation, induction of neurotrophin expression and increased adult neurogenesis (Sampedro-Piquero and Begega, 2017). Different studies have also shown that an EE ameliorates the development of neurodegenerative disorders and decreases inflammation in the brain (Laviola et al., 2008). Exposure to an EE decreases the levels of cytokines and chemokines in the hippocampus in response to lipopolysaccharide (LPS) injection (Williamson et al., 2012). An EE has also been shown to decrease the expression of inflammatory markers in microglia and brain macrophages in a model of glucocorticoid-induced depression (Chabry et al., 2015). Housing in EE conditions also decreases brain damage generated during experimental autoimmune encephalomyelitis by regulating T-cell development and function (Xiao et al., 2019).

Although most of the effects of an EE have been studied in the brain, it has also been shown to have beneficial effects outside the central nervous system, where it can alter the levels of different hormones (such as corticosterone, testosterone and oxytocin) and modulate the activation and function of the immune system in different models (Bakos et al., 2009; Marashi et al., 2003). Exposure to an EE has been shown to alter the proliferation and differentiation of T cells, B cells and natural killer (NK) cells (Gurfein et al., 2017; Meng et al., 2019; Xiao et al., 2019). An EE can also prevent immune cell senescence of macrophages, lymphocytes and NK cells, improving their function in aging mice (Arranz et al., 2010). Additionally, an EE can also have anti-tumoral effects mediated by both NK and CD8T cells (Song et al., 2017; Xiao et al., 2016). In relation to obesity, previous studies have shown that an EE prevents weight gain in a model of DIO by increasing hypothalamic brain derived neurotrophic factor (BDNF) levels, adrenergic signaling and adipose tissue browning (Cao et al., 2011).

Given this background, we sought to determine whether an EE could have beneficial effects in mice that previously presented metabolic alterations using a model of DIO. We found that housing obese mice, fed with high-fat diet (HFD), in an EE reduced fasting glucose levels and improved glucose tolerance and insulin sensitivity. EE exposure increased insulin signaling and reduced hepatic steatosis in the liver of HFD-fed mice. We also observed that an EE decreased macrophage infiltration and inflammatory mediators, but increased anti-inflammatory cytokines, lipolysis and browning markers in the adipose tissue of mice fed with HFD. Exposure to an EE also increased anorexigenic signals and decreased inflammatory proteins in the hypothalamus. These results point to a therapeutic effect of EE housing in metabolically compromised mice by alleviating inflammation in the adipose tissue and hypothalamus, thus restoring insulin signaling. Importantly, the therapeutic effect of the EE was observed even though mice were constantly fed with HFD.

## RESULTS

### EE housing ameliorates the metabolic alterations caused by a high-fat diet and improves insulin sensitivity

**Fig. 1.**
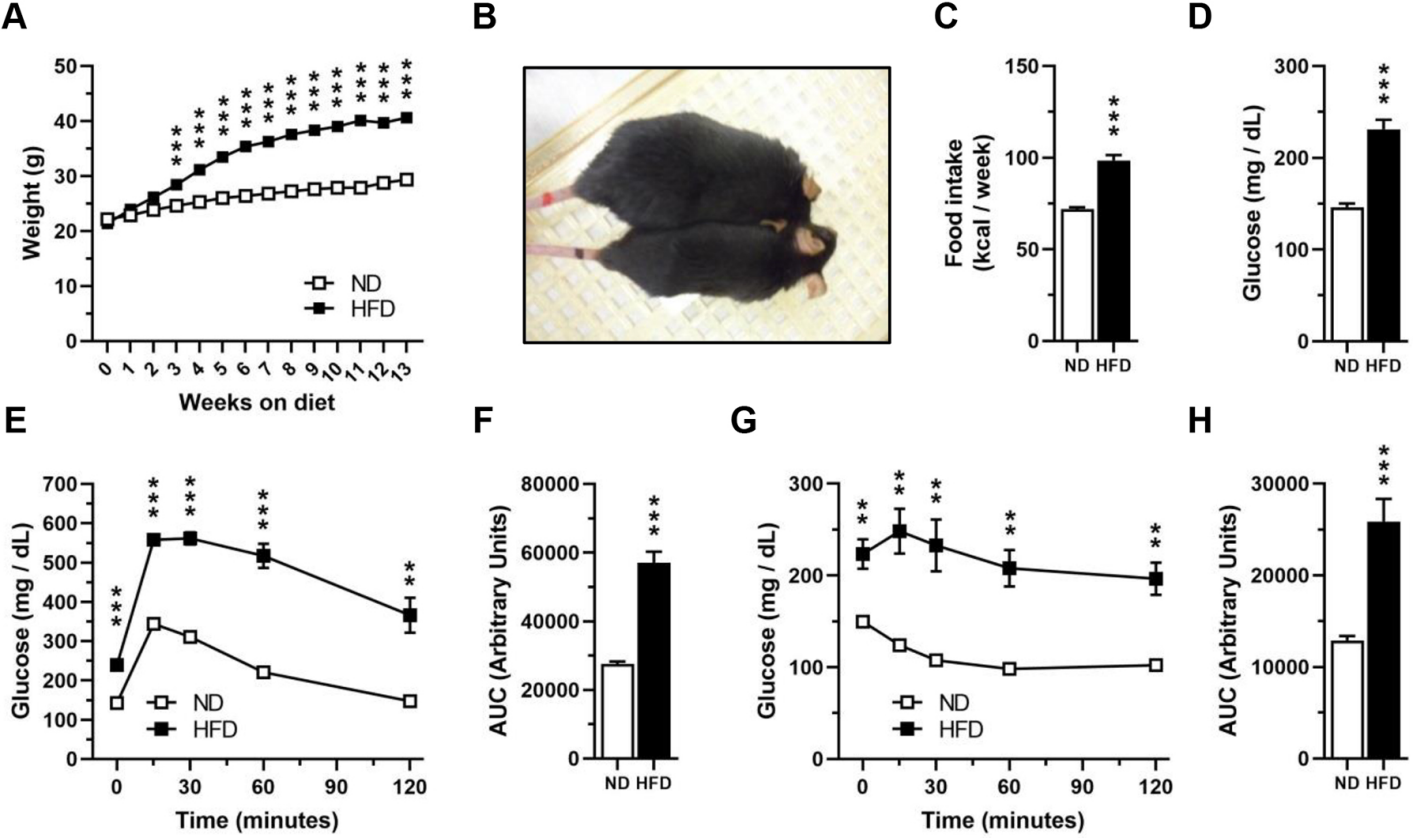
**High-fat diet feeding induces obesity and metabolic alterations in mice.** C57BL/6N mice were divided to be fed with either ND or HFD for 13 weeks in control housing conditions. (A) Average weekly weight of mice fed with ND or HFD (ND, *n*=30; HFD, *n*=44). Two-way ANOVA revealed a significant effect for interaction *F* (13, 936)=124.6, *P*<0.0001; time on diet *F* (13, 936)=499.9, *P*<0.0001; diet *F* (1, 72)=81.13, *P*<0.0001; and subject *F* (72, 936)=65.40, *P*<0.0001. (B) Representative photo of a mouse fed with ND (bottom) or HFD (top) after 13 weeks. (C) Average weekly food intake of mice fed with ND or HFD (*n*=13 weeks). Mice were fasted for 6 h to measure blood glucose levels and to perform a GTT or ITT to corroborate the effects of HFD feeding on glucose metabolism after 11 weeks of being fed with ND or HFD. (D) Fasting blood glucose levels at week 11 (*n*=20). (E) GTT after 11 weeks (*n*=10). Two-way ANOVA revealed a significant effect for interaction *F* (4, 72)=13.91, *P*<0.0001; time *F* (1.864, 33.54)=130.3, *P*<0.0001; diet *F* (1, 18)=98.57, *P*<0.0001; and subject *F* (18, 72)=5.931, *P*<0.0001. (F) AUC for the GTT (*n*=10). (G) ITT after 11 weeks (*n*=10). Two-way ANOVA revealed a significant effect for interaction *F* (4, 72)=7.337, *P*<0.0001; time *F* (2.265, 40.77)=19.33, *P*<0.0001; diet *F* (1, 18)=25.23, *P*<0.0001; and subject *F* (18, 72)=33.97, *P*<0.0001. (H) AUC for the ITT (*n*=10). Data are mean±s.e.m. **P<0.01, ***P<0.001 versus ND [two-way ANOVA followed by a Bonferroni post-hoc test (A,E,G) or unpaired *t*-test (C,D,F,H)].

C57BL/6N mice were housed in standard housing conditions and fed for 13 weeks with either a normal diet (ND), as a control group, or with HFD to generate DIO. As expected, mice fed with HFD had increased weight from week 3 to week 13 compared to those fed with ND (Fig. 1A,B), which correlated with a higher caloric intake (72±3.446 kcal/week ND group versus 98.51±10.61 kcal/week HFD group; mean±s.d.) per week (Fig. 1C). Additionally, HFD-fed mice had higher fasting glucose levels (146.2±18.25 mg/dl ND group versus 231.1±46.03 mg/dl HFD group) (Fig. 1D) and exhibited glucose intolerance (Fig. 1E,F) and insulin resistance (Fig. 1G,H), which is consistent with previous reports (Collins et al., 2004; Luo et al., 1998).

**Fig. 2.**
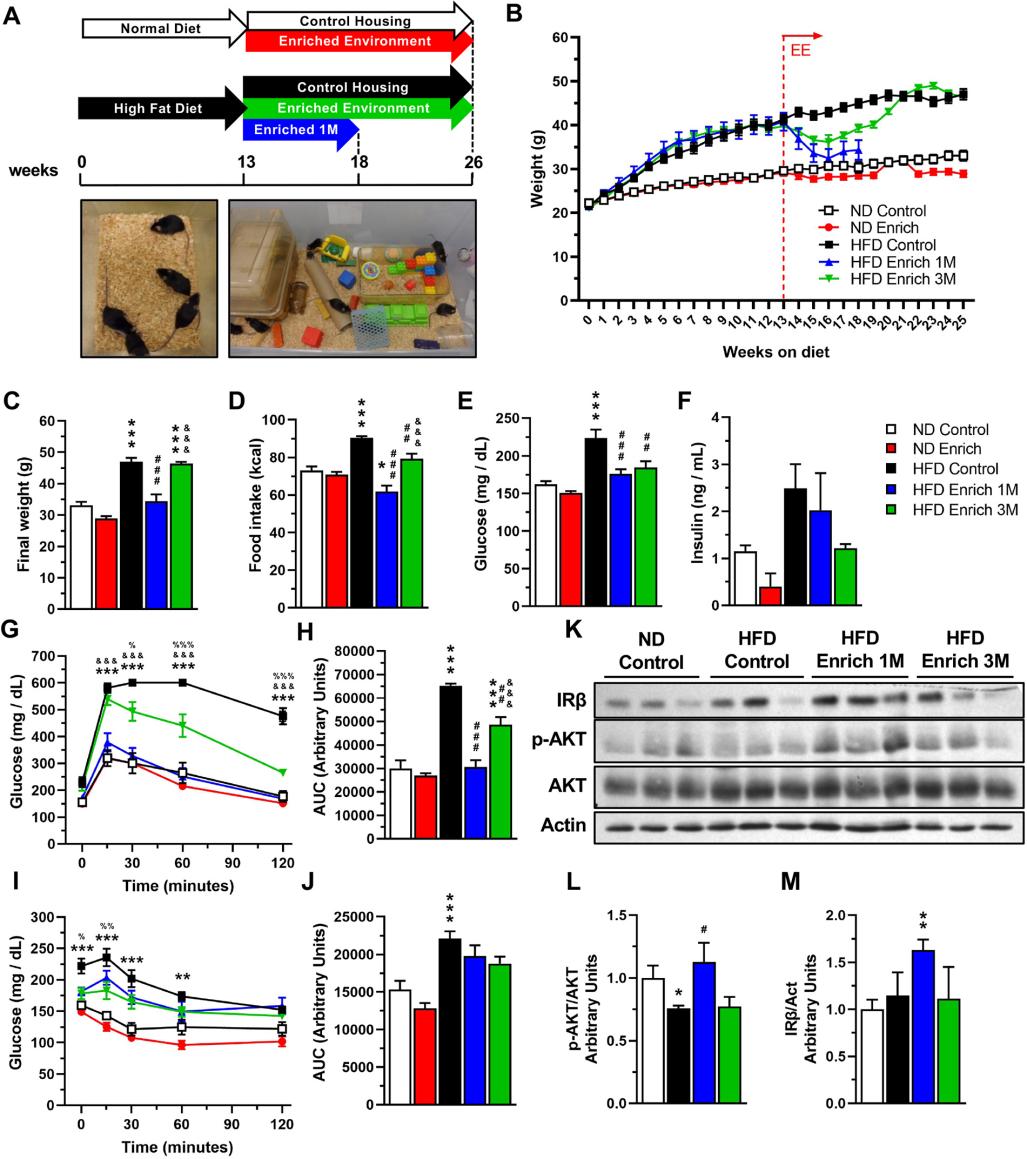
**An EE ameliorates the metabolic effects caused by a high-fat diet and improves insulin signaling in the liver.** C57BL/6N mice in control housing conditions were fed with ND or HFD for 13 weeks and were then separated into control housing or EE for an additional 5 or 13 weeks. Mice were fed with the same diet they had before they were separated into different housing conditions. Five experimental groups were formed: ND control housing (ND control); normal diet EE (ND enriched); HFD control housing (HFD control); and two HFD EE (HFD enriched) groups. Two different timepoints were used for the HFD enriched groups: HFD enriched 1 M was maintained for an additional 5 weeks, and HFD enriched 3 M was maintained for 12 weeks after the change in housing conditions together with the other control groups. (A) Experimental design. The dotted lines represent when the mice were euthanized: at 18 (for the HFD enriched 1 M) or 26 weeks (for the ND control, ND enriched, HFD control and HFD enriched 3 M groups). Representative photo of a control housing cage (left) and an EE cage (right) (not to scale). (B) Average weekly weight throughout the experiment. The dotted vertical line shows when the mice were separated into the different housing conditions at week 13 (ND control, *n*=15; ND enriched, *n*=14, HFD control, *n*=15; HFD enriched 1 M, *n*=14; HFD enriched 3 M, *n*=15). (C) Final weight at week 18 for the HFD enriched 1 M group (*n*=14), or at week 25 for the ND control (*n*=15), ND enriched (*n*=14), HFD control (*n*=15) and HFD enriched 3 M groups (*n*=15). One-way ANOVA revealed a significant difference between group means *F* (4, 68)=40.45, *P*<0.0001. (D) Weekly average of food intake after the mice were separated into different housing conditions (ND control, ND enriched, HFD control and HFD enriched 3 M, *n*=12 weeks; HFD enriched 1 M, *n*=5 weeks). One-way ANOVA revealed a significant difference between group means *F* (4, 44)=22.44, *P*<0.0001. Mice were fasted for 6 h to measure blood glucose levels and to perform a GTT or ITT to determine the effects of the housing conditions on glucose metabolism. The measurements were recorded at week 17 for the HFD enriched 1 M group or at week 25 for the ND control, ND enriched, HFD control and HFD enriched 3 M groups. (E) Fasting blood glucose levels (*n*=10). One-way ANOVA revealed a significant difference between group means *F* (4, 45)=15.28, *P*<0.0001. (F) Fasting serum insulin levels after 18 (HFD enriched 1 M) or 26 (ND control, ND enriched, HFD control and HFD enriched 3 M) experimental weeks (*n*=3). One-way ANOVA did not reveal a significant difference between group means *F* (4, 10)=3.358, *P*=0.0547. (G) GTT (*n*=5). Two-way ANOVA revealed a significant effect for interaction *F*(16, 80)=11.79, *P*<0.0001; time *F* (4, 80)=219.1, *P*<0.0001; group *F* (4, 20)=40.19, *P*<0.0001; and subject *F* (20, 80)=6.836, *P*<0.0001. (H) AUC for the GTT. One-way ANOVA revealed a significant difference between group means *F*(4, 20)=41.07, *P*<0.0001. (I) ITT (ND control, *n*=10; ND enriched, *n*=10; HFD control, *n*=9; HFD enriched 1 M, *n*=5; HFD enriched 3 M, *n*=7). Two-way ANOVA revealed a significant effect for time × group *F* (16, 144)=4.511, *P*<0.0001; time *F* (4, 144)=63.93, *P*<0.0001; group *F* (4, 36)=17.64, *P*<0.0001; and subject *F* (36, 144)=10.44, *P*<0.0001. (J) AUC for the ITT. One-way ANOVA revealed a significant difference between group means *F* (4, 36)=14.49, *P*<0.0001. (K) Representative western blot of proteins of the insulin signaling pathway in liver. (L,M) Densitometric analysis of protein levels of phosphorylated AKT Ser473 (p-AKT) (L), and insulin receptor β subunit (IRβ) (M) (*n*=6). Data are mean±s.e.m. *P<0.05, **P<0.01, ***P<0.001, versus ND control; ^#^*P*<0.05, ^##^*P*<0.01, ^###^*P*<0.001, versus HFD control; ^&&&^*P*<0.001, versus HFD enriched 1 M; and ^%^*P*<0.05, ^%%^*P*<0.01, ^%%%^*P*<0.001, versus HFD enriched 3 M [two-way ANOVA followed by a Tukey's multiple comparisons test (G,I); one-way ANOVA followed by a Tukey's multiple comparisons test (C-F,H,J); or unpaired *t*-test (L,M)]. Statistical significance against the ND enriched group is not shown on the graphs for clarity. For G and I, only comparisons against the HFD control group are shown in the graph.

To determine whether an EE is capable of ameliorating the metabolic alterations observed in these obese mice, we divided the mice so that they were kept either in standard housing conditions (ND control and HFD control groups) or in an EE (ND enriched and HFD enriched groups) (Fig. 2A). Mice were maintained on the same diet they had been given before the switch in housing conditions for an additional 3 months to evaluate weight gain, food intake and to perform metabolic tests. To elucidate whether the EE modulates the metabolic alterations in a time-dependent manner, we included a HFD group exposed to the EE for only 5 weeks before euthanasia, making two HFD enriched groups: HFD enriched 1 M and HFD enriched 3 M (Fig. 2A)

Mice fed with ND and maintained either in control housing conditions (ND control) or in EE (ND enriched) presented very similar weight throughout the experiment (final weight, 33.06±4.493 g ND control versus 28.89±2.775 g ND enriched; mean±s.d.) (Fig. 2B,C), which correlated with similar energy intake between both groups (72.93±7.414 kcal/week ND control versus 70.72±5.054 kcal/week ND enriched) (Fig. 2D). We also observed no differences in the levels of fasting glucose (162.1±13.32 mg/dl ND control versus 150.5±8.6 mg/dl ND enriched) (Fig. 2E) and serum insulin levels (1.149±0.2166 ng/ml ND control versus 0.4039±0.4755 ng/ml ND enriched) (Fig. 2F) when comparing both ND-fed groups. Accordingly, we observed that mice fed with ND had normal glucose tolerance (Fig. 2G,H) and insulin sensitivity (Fig. 2I,J), showing no difference between both housing conditions at the end of the experiment. However, comparing the data of only the ND-fed mice before the change in housing conditions (ND) with the results collected at the end of the experiment (ND control and ND enriched), we found that ND control mice gained weight (Fig. S1A, left panel) without any change in food intake (Fig. S1B, left panel) and had a small increase in fasting glucose levels (Fig. S1C, left panel), but showed similar glucose tolerance (Fig. S1D) and a diminished insulin response with aging (Fig. S1G), which was not observed in the ND enriched group. These results suggest that EE maintains metabolic homeostasis, even in ND-fed mice.

On the other hand, the HFD control group gained weight over the entire experiment (Fig. 2B). Mice that were fed with HFD and then switched to EE had an initial weight loss in both groups (HFD enriched 1 M and HFD enriched 3 M) (Fig. 2B), but surprisingly, the HFD enriched 3 M group reached the same weight as the HFD control group after 25 experimental weeks (47.03±4.727 g HFD control versus 46.38±2.25 g HFD enriched 3 M) (Fig. 2C), even though they had a lower weekly energy intake (79.24±8.85 kcal HFD enriched 3 M versus 90.38±2.71 kcal HFD control) (Fig. 2D). HFD enriched 1 M group had a lower final weight compared to both the HFD enriched 3 M and the HFD control groups (35.35±8.37 g HFD enriched 1 M) at week 18 (Fig. 2C), which correlated with lower caloric intake (61.88±6.88 kcal HFD enriched 1 M) (Fig. 2D).

Even though both the HFD control and HFD enriched 3 M groups exhibited the same weight at the end of the experiment, we identified differences in several metabolic parameters between these groups. We observed that both HFD enriched groups had lower fasting glucose levels compared to the HFD control group (226.3±36.51 mg/dl HFD control versus 176.1±18.86 mg/dl HFD enriched 1 M, and 181.7±29.34 mg/dl HFD enriched 3 M) (Fig. 2E). Still, there was no difference in serum insulin levels with respect to HFD control group (2.488±0.8924 ng/ml HFD control versus 1.217±0.1501 ng/ml HFD enriched 3 M), although insulin levels in the HFD enriched 3 M mice were similar to those observed in the ND control group (Fig. 2F). Accordingly, both HFD enriched groups had improved glucose tolerance compared to the HFD control group, with the HFD enriched 1 M group and the ND groups having a similar response in the glucose tolerance test (GTT; Fig. 2G,H). In the insulin resistance test, we found lower glucose levels in HFD enriched 3 M group compared to HFD control group (Fig. 2I). This decrement was particularly evident at the first two timepoints (T=0 and 15 min) after the insulin injection compared to the HFD control group, suggesting that mice housed in an EE for 3 months had increased insulin sensitivity (Fig. 2I). Comparing the data obtained from the HFD-fed mice before the change in housing conditions (HFD) and at the end of the experiment (HFD control, HFD enriched 1 M and HFD enriched 3 M), we observed that both HFD control and HFD enriched 3 M mice gained weight compared to HFD-fed mice at 13 weeks, whereas HFD enriched 1 M mice lost weight (Fig. S1A, right panel). Additionally, both EE groups (HFD enriched 1 M and HFD enriched 3 M) showed decreased food intake and fasting glucose levels (Fig. S1B,C, right panels), whereas the HFD enriched 1 M group had improved glucose tolerance (Fig. S1E,F, right panel) and the HFD enriched 3 M group presented higher insulin sensitivity (Fig. S1H,I, right panel) compared to the HFD-fed mice before the change in housing conditions. These data indicate that our EE conditions restore the metabolic alterations in metabolically compromised mice fed with a HFD.

To further determine the effects of EE housing on insulin sensitivity, we analyzed the activation of the insulin signaling pathway in the liver (Fig. 2K). Previous studies have shown that HFD feeding inhibits insulin signaling in different tissues by reducing AKT phosphorylation (p-AKT) (Sabio et al., 2008), an important component of the insulin signaling pathway. As expected, we observed that the administration of HFD reduced p-AKT levels in the liver compared to the ND control group (Fig. 2L). Importantly, we found that exposure to an EE increased the levels of p-AKT in the liver after 1 month in the EE (HFD enriched 1 M), but this increase was lost after a 3-month exposure to the EE (HFD enriched 3 M) (Fig. 2L). Interestingly, increased p-AKT levels correlated with higher levels of the beta subunit of the insulin receptor (IRβ) in the HFD enriched 1 M group (Fig. 2M). Together, these data indicate that an EE is capable of re-establishing insulin signaling in the liver and improving glucose metabolism in obese mice.

### An EE reduces the inflammatory process in the adipose tissue of obese mice

**Fig. 3.**
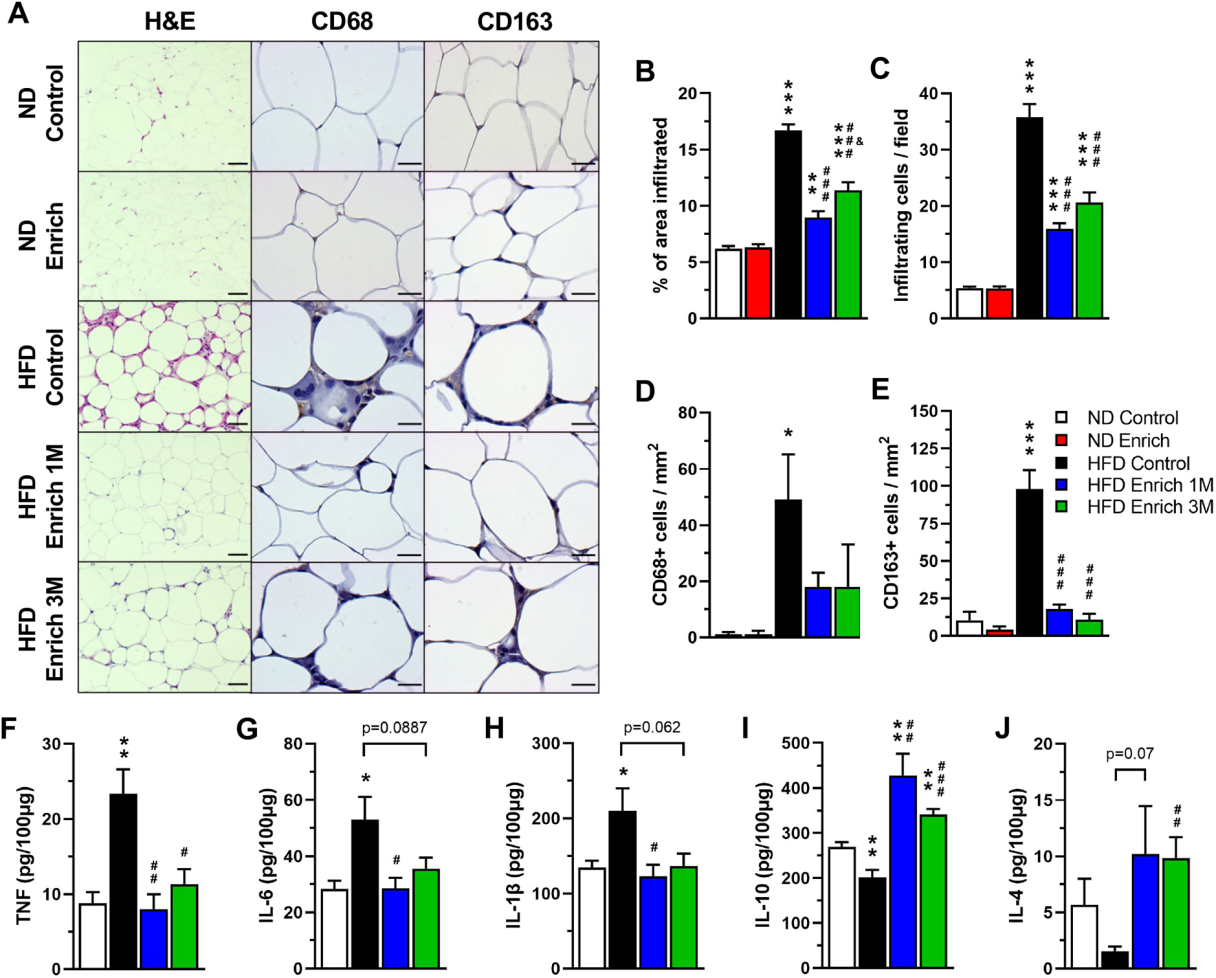
**An EE reduces cell infiltration and inflammation in the adipose tissue.** (A) Representative photos of H&E staining and immunohistochemistry for macrophage markers CD68 and CD163 in epididymal WAT. Scale bars: 200 μm for H&E; 50 μm for CD68 and CD163. (B,C) Percentage of infiltrated area (B) and infiltrating cell count (C) in WAT determined by H&E staining (*n*=5). One-way ANOVA revealed a significant difference between group means for the infiltrated area [*F* (4, 235)=66.99, *P*<0.0001] and number of infiltrating cells [*F* (4, 235)=76.38, *P*<0.0001]. (D,E) Number of infiltrating CD68^+^ cells (D) and CD163^+^ cells (E) determined by immunohistochemistry in WAT (*n*=5). One-way ANOVA did not reveal a significant difference between group means for the number of CD68^+^ cells [*F* (4, 20)=3.694, *P*=0.0208], but did reveal a significant difference for the number of CD163^+^ cells [*F* (4, 20)=34.46, *P*<0.0001]. (F-J) WAT protein levels of TNF (F), IL-6 (G), IL-1β (H), IL-10 (I) and IL-4 (J) determined by ELISA (*n*=5). Data are mean±s.e.m. *P<0.05, **P<0.01, ***P<0.001, versus ND control; ^#^*P*<0.05, ^##^*P*<0.01, ^###^*P*<0.001, versus HFD control; and ^&^*P*<0.05, versus HFD enriched 1 M [one-way ANOVA followed by a Tukey's multiple comparisons test (B-E) or unpaired *t*-test (F-J)]. Statistical significance against ND enriched is not shown on the graphs for clarity.

An excess in energy intake generates cellular stress in the adipose tissue, which is accompanied by the secretion of chemokines and cytokines that lead to the recruitment and activation of immune cells and to the development of a low-grade chronic inflammatory state in this tissue (Reilly and Saltiel, 2017). To assess whether the effect of an EE on the damage caused by DIO was associated with a reduction in the inflammatory process, we performed histological analyses of epididymal white adipose tissue (WAT) (Fig. 3A). As previously reported (Strissel et al., 2007), we observed increased cell infiltration into WAT of HFD control group compared to ND-fed mice (Fig. 3A-C). Interestingly, the area and number of infiltrated cells into WAT were decreased in both the HFD enriched 1 M and the HFD enriched 3 M groups compared to the HFD control group (Fig. 3A-C). Given that macrophage infiltration into WAT has been reported to commonly occur during obesity (Weisberg et al., 2003), we determined the identity of the infiltrated cells by immunohistochemistry assays using the CD68 and CD163 macrophage markers (Barros et al., 2013; Fig. 3A). We observed that HFD induces the recruitment of CD68^+^ and CD163^+^ macrophages to WAT compared to the ND control group (Fig. 3D,E). Interestingly, exposure to the EE significantly reduced the number of CD163^+^ macrophages and slightly diminished the number of CD68^+^ macrophages in both HFD enriched groups (Fig. 3D,E). These data indicate that EE reduces immune cell infiltration and suggest reduced inflammation in the WAT.

To gain more insight into the inflammatory process in WAT, we determined the secretion profile of different cytokines and chemokines using an antibody array (Fig. S2, Table S1). We observed that the levels of several proteins (leptin, TNF, TNFRI, TNFRII, FASL, IL-2, IL-9, IL-12 p70, CCL2, CCL3, CCL9, CCL11, CCL24, CXCL1, CX3CL1, G-CSF, M-CSF, and CD30L) were increased at least 2-fold in the HFD control group against the ND control group (Table S1). Strikingly, we found that many of these inflammatory markers were reduced in the WAT of both HFD enriched 1 M and HFD enriched 3 M mice compared to that of the HFD control mice (e.g. leptin, CCL2, CCL9, CCL11, CCL24, IL-2, IL-6, IL-12 p70, TNF, FASL, G-CSF and M-CSF, among others) (Fig. S2, Table S1). These data were further corroborated in epididymal WAT by ELISA assays. We found increased protein levels of the proinflammatory cytokines TNF (Fig. 3F), IL-6 (Fig. 3G) and IL-1β (Fig. 3H) in the WAT of HFD control mice compared to ND-fed mice. Importantly, the EE restored the levels of these inflammatory cytokines to those observed in the WAT of the animals fed with ND (Fig. 3F-H). TNF levels were significantly reduced in both EE groups compared to HFD control mice (Fig. 3F), and IL-6 and IL-Iβ were reduced in the WAT of HFD enriched 1 M mice compared to HFD control mice (Fig. 3G,H). We also found reduced levels of the anti-inflammatory cytokines IL-10 (compared to the ND control group, Fig. 3I) and IL-4 (Fig. 3J) in epididymal WAT of HFD control mice. Interestingly, IL-10 was increased in both EE groups compared to ND control and HFD control mice (Fig. 3I); in contrast, IL-4 was significantly increased in the WAT of HFD enriched 3 M mice compared to HFD control mice (Fig. 3J). Together, these results demonstrate that the EE reduces the inflammatory process in adipose tissue of HFD-fed mice favoring an anti-inflammatory status.

### An EE reduces hepatic steatosis, promotes lipolysis in the adipose tissue and induces hypothalamic anorexigenic signals

**Fig. 4.**
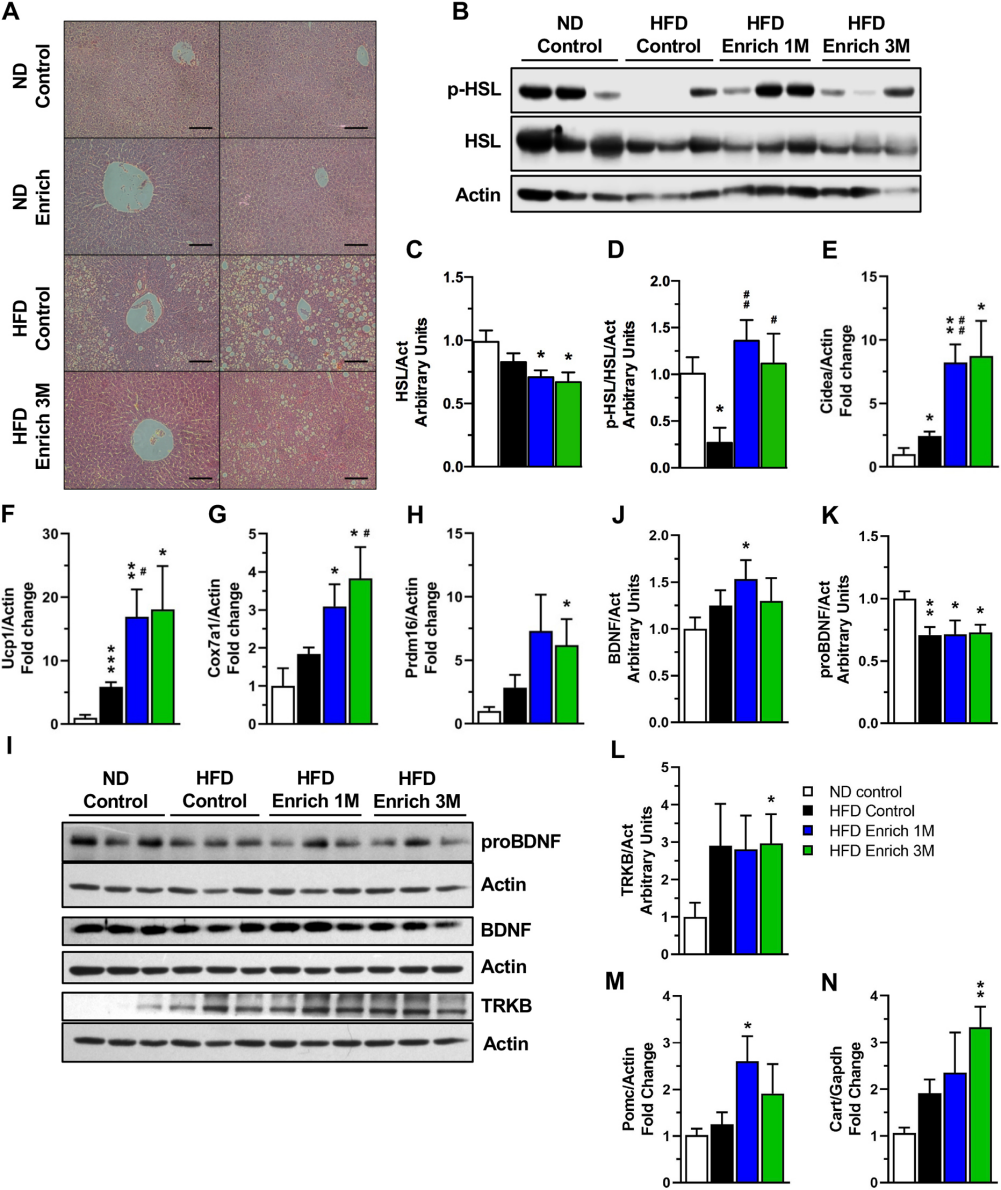
**An EE reduces hepatic steatosis, promotes white adipose tissue browning and increases anorexigenic markers in the hypothalamus.** (A) Representative photos of liver H&E staining. Scale bars: 100 μm. (B) Representative western blot of HSL activation in epididymal WAT. (C,D) Densitometric analysis of protein levels of total HSL (C) and phosphorylated HSL Ser660 (p-HSL) (D) in WAT (*n*=5). (E-H) Gene expression of browning markers *Cidea* (E), *Ucp1* (F), *Cox7a1* (G) and *Prdm16* (H) in WAT (*n*=5). (I) Representative western blot of BDNF signaling pathway components in the hypothalamus. (J-L) Densitometric analysis of hypothalamic mature BDNF (BDNF) (J), BDNF precursor (proBDNF) (K) and TRKB (L) protein levels (*n*=6). (M,N) *Pomc* (M) and *Cart* (N) gene expression in hypothalamus (*n*=3). Data are mean±s.e.m. *P<0.05, **P<0.01, ***P<0.001, versus ND control; ^#^*P*<0.05, ^##^*P*<0.01, versus HFD control (unpaired two-tailed *t*-test).

HFD feeding has been shown to promote lipid accumulation in the liver, leading to non-alcoholic hepatic steatosis (Koyama and Brenner, 2017). We found that EE housing for 3 months visibly diminished liver steatosis resulting from HFD feeding compared to HFD control mice (Fig. 4A).

WAT serves as the primary depot for lipids in the body. The hormone-sensitive lipase (HSL) is the rate-limiting enzyme in the hydrolysis of adipose-tissue-stored triglycerides (Jocken and Blaak, 2008). In response to catecholamines, HSL is phosphorylated and activated via a PKA-dependent mechanism, allowing its translocation to lipid droplets (Frühbeck et al., 2014). To further support the idea that exposure to EE improves lipid metabolism, we determined the levels of HSL phosphorylation in the WAT (Fig. 4B). We found that total HSL was reduced in the WAT of HFD enriched 1 M and 3 M mice compared to ND-fed mice (Fig. 4C). When examining HSL activation, we found that the levels of phosphorylated HSL at Ser^660^ in WAT were decreased in response to HFD feeding (Fig. 4D), as reported previously (Gaidhu et al., 2010). Even though total HSL was reduced in HFD enriched mice, the levels of phosphorylated HSL at Ser^660^ were maintained in the WAT of mice in the HFD enriched 1 M and 3 M groups compared to ND control group (Fig. 4D). These data suggest that an EE decreases lipid accumulation in the liver while maintaining fatty acid lipolysis in adipose tissue of HFD-fed mice, which may contribute to an improved metabolic state.

WAT browning has well-known benefits for whole-body energy balance, increasing energy expenditure and decreasing weight gain (Boström et al., 2012; Seale et al., 2011). Therefore, we determined whether EE housing is able to induce this process in obese mice that present metabolic alterations by measuring the expression level of different browning markers in WAT (Barneda et al., 2015; Maurer et al., 2015; Pettersen et al., 2019; Seale et al., 2011). Interestingly, exposure to an EE increased the expression of cell death-inducing DNA fragmentation factor alpha subunit-like effector A (*Cidea*), uncoupling protein 1 (*Ucp1*) and cytochrome c oxidase subunit 7A1 (*Cox7a1*) in a time-dependent manner (Fig. 4E-G). *Cidea* and *Ucp1* mRNA levels were increased after a 1-month exposure to the EE by 3.39-fold and 2.89-fold, respectively, compared to the HFD control group (Fig. 4E,F), and similar levels were maintained after 3 months in EE housing. In addition to this, 3-month exposure to an EE increased *Cox7a1* mRNA levels by 2.08-fold compared to HFD control group (Fig. 4G). Also, both HFD enriched groups presented significantly increased mRNA levels of *Ucp1*, *Cidea* and *Cox7a1* compared to ND control mice (Fig. 4E-G), whereas PR/SET domain 16 (*Prdm16*) was only significantly increased in the HFD enriched 3 M group (Fig. 4H). These results indicate that an EE induces WAT browning even in mice with obesity, which could reduce the metabolic alterations associated with HFD feeding.

An EE has been shown to promote WAT browning and to increase energy expenditure by inducing the expression of hypothalamic BDNF (Cao et al., 2011). BDNF plays an important role in energy homeostasis by having anorexigenic effects in the hypothalamus (Godar et al., 2011; Rosas-Vargas et al., 2011). Given the effects we observed in the upregulation of WAT browning markers, we examined whether under our experimental conditions the EE was also regulating BDNF signaling in the hypothalamus (Fig. 4I). Interestingly, we found increased mature BDNF protein levels in the hypothalamus of mice in the HFD enriched 1 M group (Fig. 4J). However, the unprocessed BDNF precursor (proBDNF) was reduced in all HFD-fed groups compared to ND control group (Fig. 4K). Furthermore, we observed increased protein levels of the BDNF receptor tropomyosin receptor kinase B (TRKB, also known as NTRK2) in the HFD enriched 3 M group (Fig. 4L). These results suggest that EE could enhance BDNF-TRKB signaling in the hypothalamus of HFD-fed mice.

In addition to the increased levels of hypothalamic BDNF in response to the EE, proopiomelanocortin (*Pomc*) transcript levels were significantly upregulated in the hypothalamus of the HFD enriched 1 M mice and showed a slight increase in mice exposed to the EE for 3 months (Fig. 4M). Moreover, levels of cocaine and amphetamine regulated transcript (*Cart*, also known as *Cartpt*) were also upregulated by a 3-month exposure to EE in the hypothalamus (Fig. 4N). The upregulation of BDNF, *Pomc* and *Cart* suggest increased anorexigenic signaling, which correlates with the decrease in food intake observed in both HFD enriched groups (Fig. 2D).

So far, these data indicate that an EE is able to revert the metabolic alterations caused by a HFD intake by increasing lipolysis and thermogenic gene expression in WAT, decreasing liver steatosis, and by activating hypothalamic anorexigenic signaling; processes that might lead to the improvement in glucose metabolism observed in our obese mice.

### An EE reduces inflammatory signaling in the hypothalamus of obese mice

**Fig. 5.**
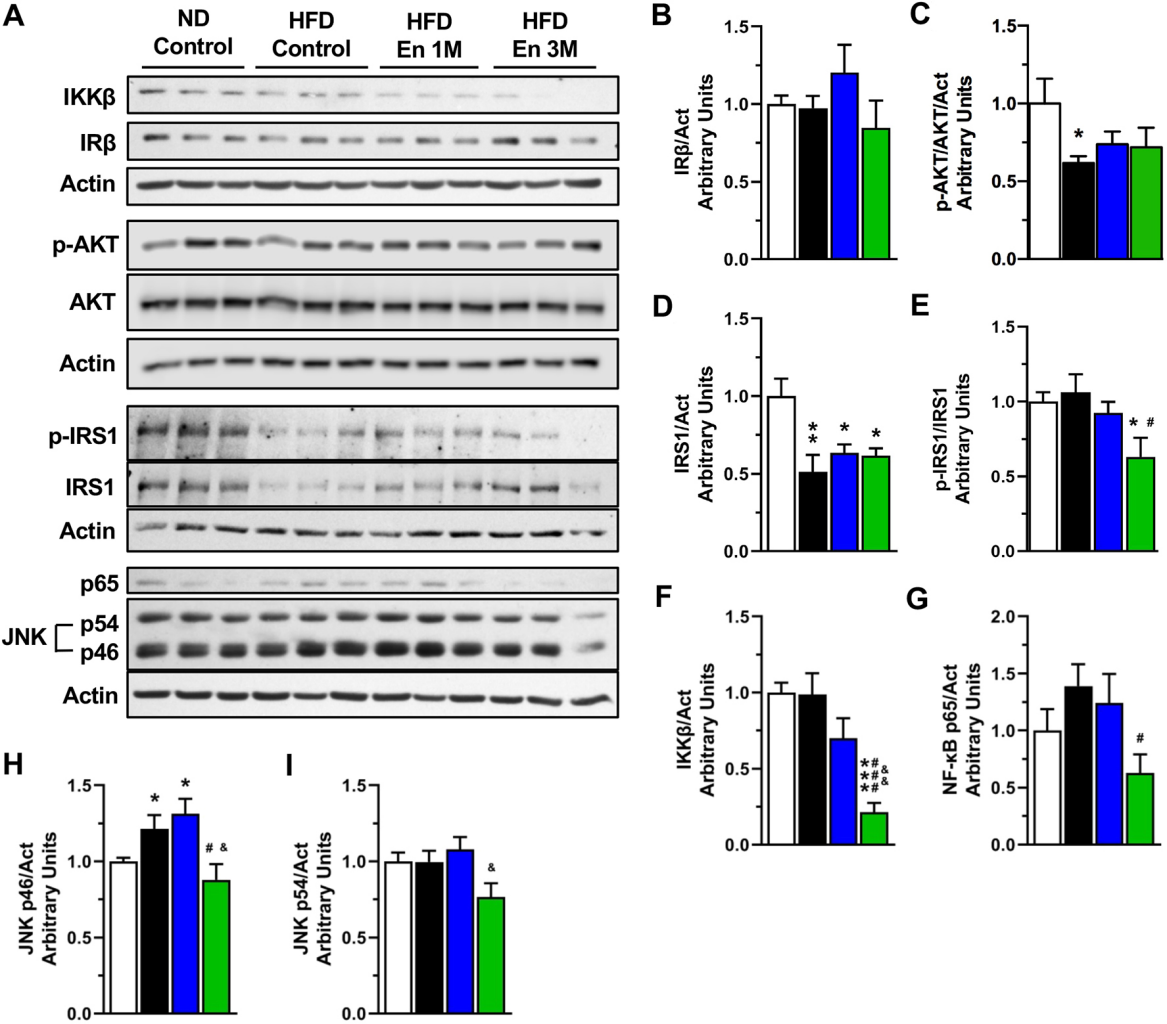
**An EE reduces hypothalamic inflammatory signaling.** (A) Representative western blot of inflammatory and insulin signaling proteins in hypothalamus. (B-I) Densitometric analysis of insulin receptor β subunit (IRβ) (B), phosphorylated AKT Ser473 (p-AKT) (C), IRS1 (D), phosphorylated IRS1 S307 (p-IRS1) (E), IKKβ (F), NF-κB subunit p65 (G), JNK p46 isoform (H) and JNK p54 isoform (I) protein levels in hypothalamus (*n*=6). Data are mean±s.e.m. *P<0.05, **P<0.01, ***P<0.001, versus ND control; ^#^*P*<0.05, ^###^*P*<0.001, versus HFD control; and ^&^*P*<0.05, ^&&^*P*<0.01, versus HFD enriched 1 M (unpaired two-tailed *t*-test).

In the brain, the hypothalamus senses energy levels to regulate food intake and energy expenditure, maintaining energy balance (Morton et al., 2006). However, many studies have observed that administration of HFD activates different inflammatory signaling pathways, such as IKK/NF-κB and JNK pathways in the hypothalamus, leading to insulin and leptin resistance (Belgardt et al., 2010; Kleinridders et al., 2009; Zhang et al., 2008). As our data show that enriched housing conditions decrease WAT inflammation, restore food intake and fasting glucose levels, re-establish glucose tolerance and improve insulin sensitivity in HFD-fed mice, we decided to evaluate the levels of certain components of the insulin signaling pathway in hypothalamus (Fig. 5A). We did not observe any change in hypothalamic levels of IRβ among all experimental groups (Fig. 5B). Even though we determined that HFD feeding decreased the levels of p-AKT compared to mice fed ND, we did not observe that an EE rescued AKT activation in the hypothalamus (Fig. 5C). Additionally, we found decreased insulin receptor substrate 1 (IRS1) levels in all mice groups fed with HFD (Fig. 5D). As the inflammatory kinases IKKβ and JNK inhibit insulin signaling via IRS-1 phosphorylation on the serine 307 residue (Aguirre et al., 2002; Gao et al., 2002), we evaluated the levels of this inhibitory IRS-1 phosphorylation (Fig. 5E). Interestingly, we found that exposure to an EE for 3 months significantly reduced the levels of phosphorylated IRS-1 Ser^307^ in the hypothalamus of HFD-fed mice compared to HFD control mice (Fig. 5E). These observations prompted us to determine the hypothalamic inflammatory status under our experimental conditions (Fig. 5A). Mice fed with HFD and housed in an EE for 3 months (HFD Enriched 3 M) showed decreased hypothalamic protein levels of the inflammatory markers IKKβ (Fig. 5F), NF-κB subunit p65 (Fig. 5G), and JNK p46 isoform (Fig. 5H), compared to HFD control mice. Levels of JNK p54 isoform were also reduced in the hypothalamus of the HFD enriched 3 M mice with respect to HFD enriched 1 M mice (Fig. 5I). Interestingly, the reduction in IRS-1 Ser^307^ phosphorylation (Fig. 5E) correlates with lower IKKβ and JNK protein levels observed in the hypothalamus of obese mice housed for 3 months in the EE (HFD enriched 3 M group) (Fig. 5F,H,I). These results indicate that our EE protocol decreases the inflammatory process in the hypothalamus induced by a HFD, even in metabolically compromised mice. Overall, our study demonstrates that a EE is able to ameliorate the metabolic alterations caused by a HFD feeding. We show that the EE re-establishes the control of energy balance by reducing inflammation in the WAT and in the hypothalamus of obese mice.

## DISCUSSION

During homeostasis, adipose tissue is in an anti-inflammatory state, in which adipocytes secrete adiponectin and macrophages show an anti-inflammatory M2 profile (Lumeng et al., 2007; Ouchi et al., 2011; Zeyda et al., 2007). Overnutrition increases the levels of inflammatory molecules, such as TNF, IL-6, CCL2 and CCL3, in adipocytes, which activate resident macrophages, leading to the recruitment of more immune cells (Ouchi et al., 2011). In addition, inflammatory signaling, mediated by cytokines or pattern recognition receptors, activate JNK and IKK kinases, which regulate insulin resistance by phosphorylating IRS, which prevents its activation by the insulin receptor (Aguirre et al., 2002; Gao et al., 2002). Using a model of DIO, we confirmed that HFD feeding increases macrophage recruitment and the levels of proinflammatory cytokines in WAT, increases lipid accumulation in liver, and leads to development of glucose intolerance and insulin resistance in mice.

Previous studies have shown that inhibiting inflammation or promoting an anti-inflammatory state in adipose tissue can improve the metabolism of obese mice. Deletion of JNK1 in adipose tissue reduces IL-6 levels, preventing insulin resistance and hepatic steatosis in a DIO model (Sabio et al., 2008). Furthermore, promoting macrophage M2 polarization decreases inflammation and increases browning markers in adipose tissue, reduces hepatic steatosis and restores glucose tolerance and insulin sensitivity in HFD-fed mice (Zhao et al., 2018). In agreement with these data, we found that exposure to an EE reduces the levels of chemokines involved in immune cells recruitment (such as CCL2, CCL5, CCL9, CCL11, CCL24 and CXCL1), as well as pro-inflammatory cytokines, such as IL-1β, TNF and IL-6, in WAT of mice fed with HFD. These data correlate with reduced infiltration of CD68^+^ and CD163^+^ macrophages in WAT of obese mice housed in our EE conditions. In accordance with these results, we have recently shown that HFD-fed mice with reduced adipose tissue inflammation have improved insulin sensitivity and glucose tolerance (Salazar-León et al., 2019), without changes in weight gain. Moreover, studies in humans have shown that obese patients with insulin sensitivity have reduced macrophage infiltration and chemokine expression in adipose tissue (Hardy et al., 2012; Klöting et al., 2010). Likewise, it is known that an EE alters the activation of the immune system, promoting an anti-inflammatory phenotype of T cells and macrophages (Chabry et al., 2015; Xiao et al., 2019). In accordance with this, we found that the EE increases the levels of IL-4 and IL-10 in WAT, which favors an M2 and anti-inflammatory milieu in adipose tissue, as described previously (Ji et al., 2012; Lumeng et al., 2007). This suggests that an EE could skew the activation state of immune cells to favor an anti-inflammatory profile in HFD-fed mice. Still, the presence of other immune cell populations and their activation state in our experimental conditions remains to be determined. Collectively, our results show that environmental enrichment could be an effective therapeutic approach against obesity-induced adipose tissue inflammation to restore glucose homeostasis.

Results regarding the effects of EE on body weight have been contradictory. Although some studies have reported that mice housed in EE and fed with ND present reduced weight (Cao et al., 2011), others have not seen such a difference (Mainardi et al., 2010; Tsai et al., 2002). In our experimental conditions, we did not find a significant difference in weight, or in other metabolic parameters, between mice fed with ND in control or EE housing conditions, which could be explained by the absence of running wheels (exercise) in our EE paradigm.

In HFD-fed mice, we found that EE caused weight loss during the first 4 weeks of the change in housing conditions. This effect was probably caused by a decrease in food intake, albeit the influence of increased physical activity might play a role in reducing weight. Nevertheless, we cannot attribute all the beneficial effects of EE to weight loss given that we observed decreased fasting glucose levels, as well as increased glucose tolerance and insulin sensitivity in obese mice (HFD enriched 3 M group). Nonetheless, we found that insulin signaling in the liver was only enhanced in the HFD enriched 1 M group, even though we observed an improvement in glucose tolerance of both HFD enriched groups (1 M and 3 M). The increase in glucose clearance observed in the HFD enriched 3 M group could suggest increased glucose effectiveness in our mice (Tonelli et al., 2005), which is known to be impaired in HFD-fed mice (Ahrén and Pacini, 2002). Previous studies have shown that weight loss in mice has rapid effects on insulin sensitivity and inflammation in liver (Schmitz et al., 2016), even though inflammation persists in adipose tissue for a longer period of time. These findings might explain the increase in insulin signaling we observed in the HFD enriched 1 M group, although other factors could also be influencing the beneficial effects of an EE in liver. In this regard, a systemic reduction in inflammation (specifically in the adipose tissue, liver and brain) has been shown to increase insulin signaling in liver (Arkan et al., 2005; Belgardt et al., 2010; Sabio et al., 2008; Yuan et al., 2001).

Most research has focused on the effect of a complete EE in different pathologies; however, other studies have tried to elucidate the contribution of different components of an EE. In some models, exercise has been shown to account for most of the beneficial effects of the EE (Brenes et al., 2016; Prado Lima et al., 2018). However, other studies have shown that the combination of different components (physical, social or cognitive stimulation) of EE provide a better effect than each component by itself (Grégoire et al., 2014; Langdon and Corbett, 2012). Accordingly, exercise alone cannot recapitulate the full effects of a complete EE on reducing adiposity or modifying gene expression in hypothalamus and in different fat depots (Cao et al., 2011). Overall, these data cannot explain how each component of an EE contributes to the beneficial effects observed in our experimental conditions, which would need further characterization.

In the adipose tissue, the inflammatory process associated with obesity is able to mediate catecholamine resistance, impairing UCP1 upregulation and HSL phosphorylation (Mowers et al., 2013). Likewise, studies in aged mice have shown that the NOD-like receptor family containing pyrin domain 3 (NLRP3)-dependent inflammation in WAT macrophages impairs catecholamine-induced lipolysis by increasing catecholamine catabolism genes (Camell et al., 2017). In our model, we observed that HFD decreased HSL phosphorylation, whereas EE maintained the levels of phosphorylated HSL, suggesting that EE sustains WAT lipolysis in obese mice. Previous studies have shown that catecholamine release by the sympathetic nervous system, and the activation of the β-adrenergic signaling pathway in adipose tissue, promotes browning (Harms and Seale, 2013). WAT browning increases energy expenditure and has beneficial effects on thermogenesis and metabolism (Boström et al., 2012; Seale et al., 2011). In our study, we found that EE increased the expression of browning markers in WAT of mice fed with HFD, including *Ucp1*. This suggests that under our experimental conditions, EE could enhance catecholamine release or re-establish catecholamine signaling in WAT to mediate the beneficial effects observed in glucose metabolism. In addition to this, catecholamines can regulate immune function and reduce macrophage inflammatory response (Barnes et al., 2015). These observations, together with our data, raise the question of whether reduced inflammation improves catecholamine signaling, or whether increasing catecholaminergic signaling reduces inflammation in WAT. Further studies are required to test these hypotheses.

Even though we observed increased HSL activation and *Ucp1* expression in the WAT of the HFD enriched 3 M group, these mice gained weight after an initial weight loss after the change in housing conditions. These results suggest that, although there is no inhibition in catecholamine signaling, energy expenditure could still be impaired. Previous studies have shown that increase in β-adrenergic-mediated cellular respiration is diminished in white adipocytes from obese patients (Yehuda-Shnaidman et al., 2010), even though lipolysis was unaffected. Furthermore, stimulation of β-adrenergic receptors in mice with a deficiency of STAT3 in adipocytes increased HSL activation and lipolysis but not fatty acid utilization and oxygen consumption (Reilly et al., 2020). Further studies are required to clarify the mechanisms regulating the improvement of glucose metabolism even in the presence of weight gain, as well as whether an EE mediates an increase in energy expenditure in HFD-fed mice.

EE has been shown to promote WAT browning by increasing norepinephrine release mediated by hypothalamic BDNF overexpression (Cao et al., 2011). Within the hypothalamus, BDNF has been shown to be part of the energy balance circuitry by regulating food intake (Bariohay et al., 2005; Xu et al., 2003). Previous studies have reported that EE increases BDNF in the brain, particularly in the hypothalamus, and that BDNF mediates many of the beneficial effects attributed to the EE (Bakos et al., 2009; Cao et al., 2011; Mainardi et al., 2010; Xiao et al., 2019). In the present study, we observed that a 1-month exposure to the EE increased mature BDNF, whereas the precursor form of BDNF was reduced. These results suggest that EE increases BDNF processing, which has been reported previously (Cao et al., 2014). However, the increase in mature BDNF levels observed in the HFD enriched 1 M group was not maintained after 3 months, but the browning markers in WAT were still increased. These data suggest that the beneficial effects of our EE protocol on the adipose tissue are mediated by different signaling pathways activated in a time-dependent manner. We propose that a short exposure to an EE induces browning markers in HFD-fed mice by a BDNF-dependent mechanism, whereas a long exposure might be independent of BDNF signaling or sustained even when BDNF levels are no longer increased.

In brain, obesity leads to an inflammatory process that impairs both insulin and leptin signaling in the hypothalamus, exacerbating obesity (Jais and Brüning, 2017). Accordingly, the inhibition of JNK or IKK/NF-κB signaling in hypothalamic neurons decreases weight gain and improves insulin signaling in models of DIO models (Belgardt et al., 2010; Benzler et al., 2013; Sabio et al., 2010). Furthermore, inhibiting inflammation can rescue learning and memory in HFD-fed mice (Lu et al., 2011). In this regard, EE has been shown to restore the cognitive impairment associated with obesity (Gergerlioglu et al., 2016), suggesting that EE might reduce the inflammatory process in the brain generated by HFD feeding. EE has also been shown to decrease IL-1β and CD68 expression in the hippocampus, as well as prevent cognitive decline in aged mice, even in the absence of running wheels (Birch and Kelly, 2019). In the present study, we found that exposure to an EE for 3 months significantly decreased JNK, IKK and NF-κB protein levels in the hypothalamus of HFD-fed mice, which further indicates that an EE is capable of reducing inflammation in the hypothalamus.

Hypothalamic IKK/NF-κB activation in response to dietary obesity induces neurodegeneration and inhibits neurogenesis, affecting neuronal phenotypes associated with energy balance, including the anorexigenic proopiomelanocortin (POMC) neurons (Li et al., 2012; Moraes et al., 2009). Congruently, the implantation of hypothalamic stem cells with impaired IKK/NF-κB signaling in HFD-fed mice increases neurogenesis and POMC neuronal differentiation, and reduces body weight, food intake, glucose intolerance and insulin levels (Li et al., 2014). The anorexigenic neurohormone CART has also been implicated in feeding behavior and energy expenditure (Lau et al., 2018). CART colocalizes with POMC in the arcuate nucleus of the hypothalamus, where *Cart* mRNA levels are increased following feeding or increased leptin levels (Elias et al., 1998; Germano et al., 2007). Interestingly, here, we report that *Pomc* and *Cart* transcript levels were significantly upregulated in HFD-fed mice exposed to EE for 1 month and 3 months, which correlates with a decrease in food intake, glucose intolerance and blood glucose levels in these mice. Therefore, further experiments are required to determine whether EE regulates *Pomc* and *Cart* expression in different hypothalamic nuclei. These data also raise the question as to whether an EE promotes neuronal regeneration, leading to increased anorexigenic signaling as part of the mechanism to regulate glucose metabolism in obese mice.

There is ample evidence about the beneficial effects of EE in brain physiology, cognition and in different disease models in both mice and rats. However, transferring what is known in murine models to treat patients has proven challenging. Some studies have managed to translate the different components of an EE (including physical, cognitive, social and somatosensorial stimulation) into clinical settings to treat stroke, neurodevelopmental disorders, age-related cognitive decline and neurodegenerative diseases (Ball et al., 2019; Clemenson et al., 2020; McDonald et al., 2018; Pradhan, 2019). In recovering stroke patients, an EE has been shown to increase engagement in physical, social or cognitive activities, improve motor performance and diminish depression and anxiety levels (Janssen et al., 2014; Rosbergen et al., 2017; Vive et al., 2020). Furthermore, an EE can improve cognition and ameliorate autism symptoms in children with autism (Woo and Leon, 2013; Woo et al., 2015). Based on the present study showing the anti-inflammatory properties of our EE protocol, it is important to consider the clinical application of an EE to treat obesity-associated inflammation to prevent the development of other pathologies, such as cardiovascular and neurodegenerative diseases, cancer and type II diabetes. The design of sensory therapy rooms using similar components of an EE as those described previously (Ball et al., 2019; Clemenson et al., 2020; McDonald et al., 2018; Pradhan, 2019), including physical, cognitive (e.g. playing an instrument, reading, listening to a podcast, crosswords, puzzles, writing and games), social (e.g. communal socialization and group activities) and somatosensorial (e.g. crafts, singing, stimulation and bouncing a ball) stimulation, in clinical settings should be considered as a potential intervention to complement current treatment options and lifestyle changes (Glechner et al., 2018) used to treat the metabolic alterations in obese/diabetic patients.

## MATERIALS AND METHODS

### Animals

C57BL/6N male mice (6-7 weeks old; Envigo) were randomized into standard housing conditions (21 cm width×29 cm long×16 cm height per cage, with 5 mice per cage) and fed with either regular chow diet (ND, 30 mice) (2018SX; Envigo Teklad Global) or with HFD (44 mice) (Research Diets, D12492). After 13 weeks, mice were divided to be either maintained in the control housing conditions or to be switched to an EE with the same diet, forming five experimental groups: ND control housing (ND control, 15 mice); ND EE (ND enriched, 15 mice); HFD control housing (HFD control, 15 mice); HFD EE 1 month (HFD enriched 1 M, 14 mice); and HFD EE 3 months (HFD enriched 3 M, 15 mice). The mice were distributed between housing condition groups in a way that minimized the difference in mean weight (for each diet). The EE housing conditions consisted of large cages (32 cm width×88 cm long×47.6 cm height per cage; 15 mice per cage) supplemented with plastic tunnels, wood and plastic toys, cardboard boxes and glass jars. The toys and their locations were changed once a week. Mice were maintained on a normal 12 h/12 h light/dark cycle with the corresponding diet and water *ad libitum*. The body weight of each mouse was recorded weekly. Food intake was recorded as the total food consumption of each cage housing five (control) or 15 (enriched) mice, and was represented as the average food intake per mouse per week. Metabolic tests were performed at week 17 for the HFD enriched 1 M mice and at week 25 for the other groups. The animals were sacrificed by CO_2_ inhalation after 18 (HFD enriched 1 M) or 26 (ND control, ND enriched, HFD control and HFD enriched 3 M) weeks on the experimental settings; blood and tissue were harvested for further use. The Institutional Bioethical Committee approved all animal experiments described in this study.

### Glucose and insulin tolerance test

A glucose tolerance test (GTT) and an insulin tolerance test (ITT) were performed at different timepoints during our experiments to evaluate the effect of diet and environmental conditions on glucose metabolism. Twenty mice from each diet group were selected after 11 weeks of ND or HFD feeding, and divided between the GTT and ITT so that their mean weight was similar between the same diet group (*n*=10). After the separation in environmental conditions, the GTT and ITT were performed after 17 experimental weeks for the HFD enriched 1 M mice (*n*=5), or after 25 experimental weeks for the other groups (ND control, ND enriched, HFD control and HFD enriched 3 M).

For the GTT and ITT, animals were fasted for 6 h leading up to the test from 8:00 to 14:00. Each mouse received an intraperitoneal injection with glucose (1.8 gr/kg) or insulin (Humulin R, 1 U/kg) for the glucose or insulin resistance test, respectively. The concentration of blood glucose was measured using a glucometer (Accu-Chek, Roche) at 0, 15, 30, 60 and 120 min post-injection.

### Area under the curve

The area under the curve (AUC) for both the ITT and GTT was calculated using Tai's formula (Tai, 1994):

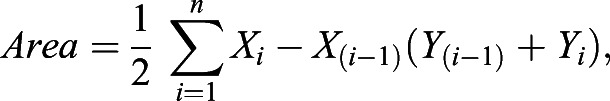
where *X*=time (0, 15, 30, 60, 120 min), and *Y*=glucose (mg/dl) levels at each timepoint.

### Tissue preparation, serum harvesting and biomarker measurements

Tissues were fixed in 4% paraformaldehyde in PBS and stored at 4°C or at −70°C until use. Blood samples, obtained by cardiac puncture, were incubated at 4°C for 2 h to promote clot formation, and were then spun at 135 ***g*** for 10 min. The serum was then collected and stored at −70°C until use.

The sera from three mice of each group was pooled before performing biomarker measurements. Circulating insulin levels were quantified in blood sera by ELISA using the Mouse Insulin ELISA Kit ALPCO (80-INSMS-E01) following the manufacturer's protocols (*n*=3 serum pools per group).

### Adipose tissue immunohistochemistry

Epididymal WAT was collected, fixed using 4% paraformaldehyde, and embedded in paraffin. Two 1-µm-thick sections were prepared, deparaffinized in xylene, rehydrated in graded ethanol series and then stained with Hematoxylin and Eosin (H&E). Analysis of adipocyte histology was performed using ImageJ software according to the manual procedure (Abràmoff et al., 2004). Immune cell infiltration in the adipose tissue was quantified by calculating the infiltration ratio of ten fields (10×) from three slides of each individual mouse, with a total of five mice per group. Light microscope images were acquired using a Nikon Eclipse Ci-L microscope with an Infinity1 Lumenera color camera.

Immunohistochemistry was performed on 2-µm-thick sections deparaffinized in xylene and rehydrated in a graded ethanol series. Antigen retrieval was performed by immersing the slides in antigen retrieval buffer [100× citrate buffer (pH 6); Abcam, ab93678] for 25 min in boiling water. Endogenous peroxidase activity was inhibited by 3% H_2_O_2_-methanol treatment for 20 min, and background non-specific binding was reduced by incubating with 1% fetal bovine serum in 1× PBS (pH 7.4) for 30 min. Sections were incubated overnight at room temperature with anti-CD163 (1:100 dilution, Invitrogen, 14-1631-82) or anti-CD68 (1:150 dilution, Invitrogen, 14-0681-82) antibodies. Goat anti-rat horseradish peroxidase (HRP) secondary antibody (HRP polymer, Abcam, ab214882) was then added for 40 min at room temperature. The slides were then washed five times in 1× PBS (pH 7.4), and incubated for 30 min at room temperature with streptavidin-HRP. The antigen-antibody complex was visualized using DAB chromogen (Abcam, ab64238). After stopping the reaction with distilled water, the sections were counterstained with Hematoxylin, washed in distilled water for 5 min and dehydrated sequentially in 70%, 90% and 100% ethanol, ethanol/xylene and xylene. The stained sections were visualized using a Nikon Eclipse Ci-L microscope with an Infinity1 Lumenera color camera and analyzed using Image-Pro Express version 6.0 software (Media Cybernetics). Ten randomly selected images per slide from five individual mice per group were taken at 20× magnification.

### Liver histochemistry

Liver tissue was mounted in Tissue-Tek optimal cutting temperature compound (Sakura Finetek) and frozen for sectioning. Liver slides were stained with H&E. Ten images, randomly selected per slide from three individual mice per group, were taken at 20× magnification to visualize lipid droplets. Light microscopic images were acquired using a Zeiss LSM510/UV Axiovert 200 M confocal microscope with a Nikon Coolpix 5000 camera.

### Protein extracts

Frozen epididymal WAT was cut into pieces in 400-600 µl lysis buffer [20 mM Tris (pH 7.4), 137 mM NaCl, 25 mM β-glycerophosphate (pH 7.4), 2 mM PPiNa, 2 mM EDTA (pH 7.4), 1% Triton X-100 and 10% glycerol]. Samples were incubated on ice for 1 h, and then centrifuged at 19,700 ***g*** at 4°C; the supernatants were recovered and stored at −70°C. Total protein extracts were quantified by the bicinchoninic acid method.

For liver and hypothalamus, samples were thawed in 300-500 µl of lysis buffer [20 mM Tris (pH 7.4), 137 mM NaCl, 25 mM β-glycerophosphate (pH 7.4), 2 mM PPiNa, 2 mM EDTA (pH 7.4), 1% Triton X-100 and 10% glycerol] supplemented with 1× complete protease inhibitor cocktail (Roche) and phosphatase inhibitors (200 mM Na_3_VO_4_, 0.1 mM dithiothreitol and 1 mM phenylmethylsulfonyl fluoride). The tissue was sonicated and the homogenate was incubated on ice for 10 min. Samples were spun at 14,500 rpm at 4°C, and supernatants were recovered and stored at −70°C. Total protein extracts were quantified using the Bradford method.

### Western blotting

Proteins (15-30 µg) were resolved on polyacrylamide gels and transferred to a nitrocellulose membrane (Hybond-ECL, GE Healthcare Life Sciences). The membrane was blocked with 5% skimmed milk dissolved in TBS-T [20 mM Tris (pH 7.5), 150 mM NaCl, 0.1% Tween 20] at room temperature for 1 h, and finally incubated with the corresponding primary antibody at 4°C overnight. The secondary antibody coupled to HRP was incubated for 1 h at room temperature in 5% milk in TBS-T or 5% bovine serum albumin in TBS-T. After washing, immune complexes were visualized using chemiluminescence with ECL Western Lighting detection reagents following the manufacturer's instructions. Densitometry analysis was performed using a Gel-Doc XR+ (Bio-Rad) and Image Lab version 6.0.1 software (Bio-Rad), or using a C-DiGit 3600 Blot Scanner (LI-COR Biosciences) and Image Studio Software version 5.2.5 (LI-COR Biosciences).

The following antibodies were used: phosphorylated AKT (p-AKT Ser473, 1:1000 dilution; Cell Signaling Technology, CS-9271); AKT (1:3000 dilution; Cell Signaling Technology, CS-9272); insulin receptor β (IRβ, 1:1000 dilution; Cell Signaling Technology, CS-3020); JNK (1:2000 dilution; Cell Signaling Technology, CS-9252); IKKβ (1:1000 dilution; Cell Signaling Technology, CS-2370); NF-κB p65 (1:1000 dilution; Cell Signaling Technology, CS-4764); IRS-1 (1:1000 dilution; Cell Signaling Technology, CS-2382); phosphorylated IRS (p-IRS Ser307, 1:1000 dilution; Cell Signaling Technology, CS-2381); BDNF (recognizes proBDNF and BNDF, 1:1000 dilution; Santa Cruz Biotechnology, sc-546); TRKB (1:1000 dilution; Santa Cruz Biotechnology, sc-8316); HSL (1:1000 dilution; Cell Signaling Technology, CS-4107); phosphorylated HSL (p-HSL Ser660, 1:1000 dilution; Cell Signaling Technology, CS-4126); and actin (1:6000 dilution; Santa Cruz Biotechnology, sc-1616).

### ELISA

Cytokine levels in the epididymal WAT were determined from total protein extracts using the IL-1β (432605), IL-4 (431101), IL-6 (431304), IL-10 (431414) and TNF (430904) ELISA MAX Deluxe Sets (BioLegend) following the manufacturer's instructions.

### Adipose tissue inflammatory profile

Protein extracts from the epididymal WAT were prepared as described above. The adipose tissue inflammatory profile was determined using the mouse cytokine antibody array C1 RayBio C-Series (AAM-INF-1-4). Arrays were probed with 250 µg of protein extracts following the manufacturer's instructions. The antibody-antigen interactions were visualized using chemiluminescence with a LI-COR Biosciences instrument. Densitometry was performed using Image Studio Software version 5.2.5.

### RNA extraction, reverse transcription and qPCR

Total RNA was isolated from epididymal WAT TRIzol reagent (Thermo Fisher Scientific, 15596026) following the manufacturer's instructions except for two additional steps. First, before adding isopropanol, TRIzol:sample homogenate was briefly centrifuged for 1 min at 15,800 ***g*** and the top lipid layer was discarded from all samples. Second, an extra 100% ethanol wash was performed before the regular 70-75% ethanol washes, as suggested by the product's protocol. RNA concentration was determined using a Nanodrop 2000 (Thermo Fisher Scientific). Total RNA (500 ng) of each sample was retrotranscribed using oligo dT, random hexamers (Thermo Fisher Scientific, N8080127) and the thermostable M-MLV reverse transcriptase (Thermo Fisher Scientific, 28025013) following the manufacturer's directions. At the end of the reverse transcription reaction, 20 µl of cDNA was diluted 1:10 in water. Finally, 1 µl of each diluted cDNA was used for qPCR assays according to the instructions from the Maxima SYBR Green/ROX 2X qPCR Master Mix kit (Thermo Fisher Scientific, K0221) with a temperature of 60°C using a StepOne Real-Time PCR system (Thermo Fisher Scientific, 4376357).

For the hypothalamus, total RNA was isolated as described previously (Pérez-Martínez et al., 1998) pooling two hypothalami for each sample. Hypothalamic RNA (1 μg) was retrotranscribed using oligo dT and M-MLV reverse transcriptase (Thermo Fisher Scientific, 28025013) following the manufacturer's directions.

Primers were designed to specifically recognize and amplify mouse genes in the qPCR assays. All the primers used in this study are listed in Table S2.

### Statistical analyses

Data are presented as mean±s.e.m. An unpaired two-tailed *t*-test was used to compare group pairs. Data were also analyzed by one-way ANOVA followed by a Tukey's post-hoc test, and two-way ANOVA followed by Tukey'multiple comparison test or Bonferroni post-hoc test. *P*<0.05 was considered statistically significant. Statistical significance tests were performed using GraphPad Prism version 9.0 for Windows.

## Supplementary Material

Supplementary information
