# Supplementary material for: An enriched environment re-establishes metabolic homeostasis by reducing obesity-induced inflammation
Source: Dis Model Mech. 2022 Jun 13;15(6):dmm048936. doi: 10.1242/dmm.048936 (PMC9227715; doi:10.1242/dmm.048936)
Supplement: Supplementary information [file dmm-15-048936-s1.pdf]

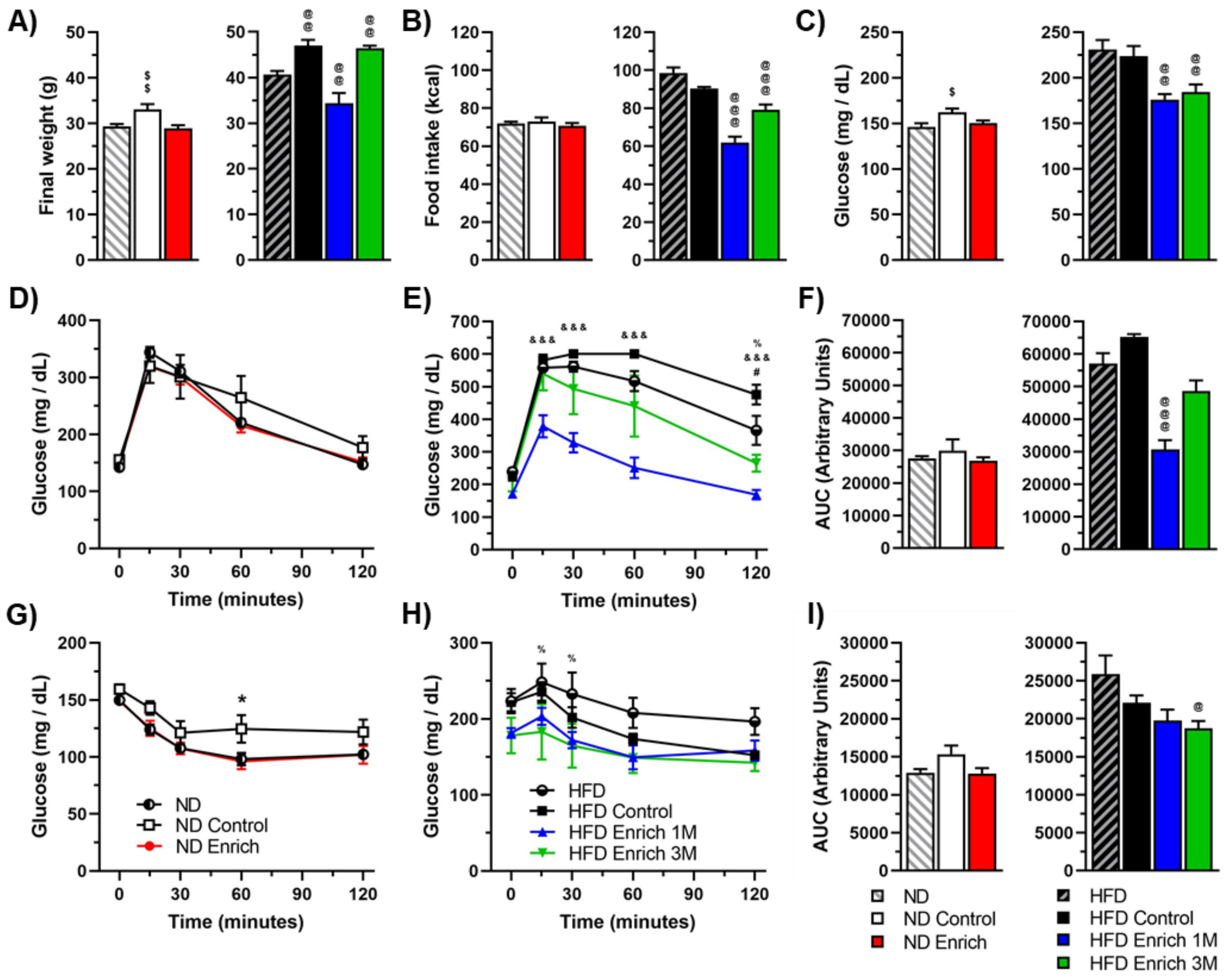

Fig. S1. Comparison between groups before the change in environmental conditions and at the end of the experiment.

Mice were fed for 13 weeks with normal (ND) or high-fat diet (HFD) and kept in control housing conditions. After this, mice were fed with the same diet they had before they were separated into different housing conditions. Five experimental groups were formed: normal diet control housing (ND Control), normal diet enriched environment (ND Enrich), high-fat diet control housing (HFD Control) and 2 high-fat diet enriched environment (HFD Enrich) groups. Two different time points were used for the HFD enriched groups: HFD Enrich 1 M was maintained for an additional 5 weeks, while the HFD Enrich 3 M was maintained for 12 weeks after the change in housing conditions together with the other groups (ND Control, ND Enrich and HFD Control). Final weight (n=14-44 mice) **(A)** and

Disease Models & Mechanisms • Supplementary information

average weekly food intake (n=5-13 weeks) **(B)** for mice groups fed with ND (left panels) or HFD (right panels). Data in these graphs represent the values obtained at 13 weeks for ND and HFD groups, 17 weeks for HFD Enrich 1 M group, and 25 weeks for ND Control, ND Enrich, HFD Control and HFD Enrich 3 M groups.

Mice were fasted for 6 hours to measure blood glucose levels and to perform glucose tolerance test (GTT) or insulin tolerance test (ITT). **C)** Comparison of fasting blood glucose levels for mice fed with ND diet (left panel) and HFD diet (right panel) (n=10-20). GTT for mice groups fed with ND **(D)** or HFD **(E)** (n=5-10). **F)** Area under the curve for the GTT for the mice groups fed with ND (left panel) or HFD (right panel). ITT for the mice groups fed with ND **(G)** or HFD **(H)** (n=5-10). **I)** Area under the curve for GTT for the mice groups fed with a ND (left panel) or a HFD (right panel). The data presented in **(C-I)** represent the values obtained at 11 weeks for the ND and HFD groups, 17 weeks for the HFD Enrich 1 M group, and 25 weeks for the ND Control, ND Enrich, HFD Control and HFD Enrich 3 M groups. All graphs represent mean ± s.e.m. * $P<0.05$, ** $P<0.01$, *** $P<0.001$. For (A, B, C, F, and I) comparisons were done between groups of the same diet against the groups before the change in housing condition and statistics were determined by one-way ANOVA followed by a Tukey's multiple comparisons test, $ vs ND, @ vs HFD. For (D and G) statistics are shown against the ND group before the change in housing conditions determined by two-way ANOVA followed by a Tukey's multiple comparisons test, * vs ND Control, + vs ND Enrich. For (E and H) statistics are shown against the HFD group before the change in housing conditions determined by two-way ANOVA followed by a Tukey's multiple comparisons test, # vs HFD Control, & vs HFD Enrich 1 M and % vs HFD Enrich 3 M.

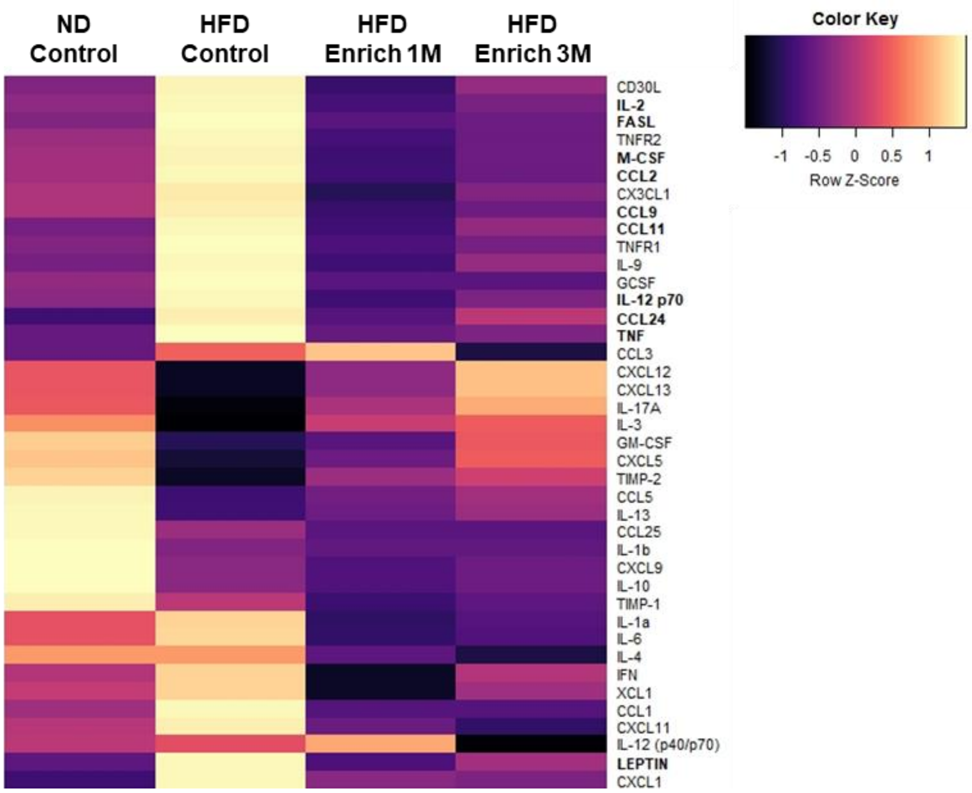

Fig. S2. An enriched environment reduces cytokine and chemokine levels in the white adipose tissue of obese mice.

Inflammatory protein levels in epididymal white adipose tissue were determined by an antibody array. The groups tested were: normal diet (ND) control housing (Control), high-fat diet (HFD) control (HFD Control), HFD enriched environment 1 month (Enrich 1 M) and HFD Enrich 3 M. Protein levels were normalized against ND Control (n=1 mouse per group).

Table S1. An enriched environment reduces cytokine and chemokine levels in the white adipose tissue of obese mice

Molecule	ND Control	HFD Control	HFD Enrich 1M	HFD Enrich 3M
LEPTIN	1	4.8	0.8	1.9
CXCL1	1	3.5	1.6	1.5
IL-12 p70	1	3.3	0.3	0.9
CCL11	1	2.9	0.6	1.2
TNFR1	1	2.9	0.6	0.9
G-CSF	1	2.7	0.6	0.6
IL-9	1	2.6	0.7	1.2
CCL24	1	2.5	1.1	1.6
CCL9	1	2.5	0.1	0.5
CD30L	1	2.3	0.6	1.1
CX3CL1	1	2.3	0.1	0.7
IL-2	1	2.3	0.6	0.9
M-CSF	1	2.3	0.4	0.7
TNFR2	1	2.3	0.5	0.7
FASL	1	2.2	0.8	0.9
CCL2	1	2.1	0.5	0.7
TNF	1	2.1	1	1.1
CCL3	1	2	2.6	0.5
CXCL11	1	1.7	0.7	0.5
CCL1	1	1.6	0.8	0.8
IL-6	1	1.4	0.4	0.5
XCL1	1	1.4	0.5	0.9
IFNγ	1	1.3	0.7	1
IL-1α	1	1.3	0.5	0.6
IL-12 (p40/p70)	1	1.1	1.3	0.5
IL-4	1	1	0.7	0.6
CXCL13	1	0.5	0.8	1.2
CXCL12	1	0.5	0.8	1.2
GM-CSF	1	0.4	0.5	0.8
CCL5	1	0.4	0.5	0.6
TIMP-1	1	0.4	0	0.1
IL-3	1	0.3	0.8	0.9
IL-13	1	0.3	0.4	0.5
IL-17A	1	0.3	0.8	1.2
CXCL5	1	0.3	0.5	0.8
CCL25	1	0.3	0.1	0.1
TIMP-2	1	0.3	0.6	0.7
IL-1β	1	0.2	0.1	0.1
IL-10	1	0.2	0	0.1
CXCL9	1	0.2	0	0.1

Disease Models & Mechanisms • Supplementary information

Raw data for supplementary figure 2. Inflammatory protein levels in epididymal white adipose tissue were determined by an antibody array. Groups tested were: normal diet (ND) control housing (Control), high-fat diet (HFD) Control, HFD enriched environment 1 month (Enrich 1 M) and HFD Enrich 3 M. Protein levels were normalized against ND Control (n=1 mouse per group).

Table S2. qPCR primers used in this study

Gene name	Transcript ID	Forward	Reverse	Amplicon size (bp)
Actb	ENSMUST00000100497.10	CTAAGGCCAACCGTGAAAAG	CATCACAATGCCTGTGGTAC	125
Prdm16	ENSMUST00000030902.12	AGCTGAGGAAGCATTTGAAGT	CGTGGAGAGGAGTGTCTTC	140
Ucp1	ENSMUST00000034146.4	CTCTCTGCCAGGACAGTAC	GCTGTTCAAAGCACACAAAC	149
Cidea	ENSMUST00000025404.9	GGAAAAGGGACAGAAATGGAC	CGTGGCTTTGACATTGAGAC	145
Cox7a1	ENSMUST00000098594.3	GGCAGAGAAGCAGAAGCTC	CCAGCCCAAGCAGTATAAGC	142
Pdk4	ENSMUST00000019721.6	GTCAGGTTATGGGACAGACG	CCTGCTTGGGATACACCAG	142
Cart	ENSMUST00000022150.7	AGAAGAAGTACGGCCAAGTCC	CACACAGCTTCCCGATCC	84
Pomc	ENSMUST00000020990.7	CTCCTGCTTCAGACCTCC	CAGTCAGGGGCTGTTCAT	169
Gapdh	ENSMUST00000073605.14	CATTGTGGAAGGGCTCATGA	GGAGGCCATGCCAGTGAGC	193

Cart primer sequences were obtained from (Konieczna et al., 2013).

References

Konieczna, J., García, A. P., Sánchez, J., Palou, M., Palou, A. and Picó, C. (2013). Oral leptin treatment in suckling rats ameliorates detrimental effects in hypothalamic structure and function caused by maternal caloric restriction during gestation. *PLoS One* **8**, e81906.

Disease Models & Mechanisms • Supplementary information
